# Transcriptomic and Bioinformatic Analyses Identifying a Central Mif-Cop9-Nf-kB Signaling Network in Innate Immunity Response of *Ciona robusta*

**DOI:** 10.3390/ijms24044112

**Published:** 2023-02-18

**Authors:** Laura La Paglia, Mirella Vazzana, Manuela Mauro, Francesca Dumas, Antonino Fiannaca, Alfonso Urso, Vincenzo Arizza, Aiti Vizzini

**Affiliations:** 1 Istituto di Calcolo e Reti ad Alte Prestazioni-Consiglio Nazionale delle Ricerche, Via Ugo La Malfa 153, 90146 Palermo, Italy; 2 Dipartimento di Scienze e Tecnologie Biologiche, Chimiche e Farmaceutiche-Università di Palermo, Via Archirafi 18, 90128 Palermo, Italy

**Keywords:** LPS, *Ciona robusta*, transcriptome, innate immunity, cytokine, miRNA

## Abstract

The Ascidian *C. robusta* is a powerful model for studying innate immunity. LPS induction activates inflammatory-like reactions in the pharynx and the expression of several innate immune genes in granulocyte hemocytes such as cytokines, for instance, macrophage migration inhibitory factors (CrMifs). This leads to intracellular signaling involving the Nf-kB signaling cascade that triggers downstream pro-inflammatory gene expression. In mammals, the COP9 (Constitutive photomorphogenesis 9) signalosome (CSN) complex also results in the activation of the NF-kB pathway. It is a highly conserved complex in vertebrates, mainly engaged in proteasome degradation which is essential for maintaining processes such as cell cycle, DNA repair, and differentiation. In the present study, we used bioinformatics and in-silico analyses combined with an in-vivo LPS exposure strategy, next-generation sequencing (NGS), and qRT-PCR to elucidate molecules and the temporal dynamics of Mif cytokines, Csn signaling components, and the Nf-κB signaling pathway in *C. robusta*. A qRT-PCR analysis of immune genes selected from transcriptome data revealed a biphasic activation of the inflammatory response. A phylogenetic and STRING analysis indicated an evolutionarily conserved functional link between the Mif-Csn-Nf-kB axis in ascidian *C. robusta* during LPS-mediated inflammation response, finely regulated by non-coding molecules such as microRNAs (miRNAs).

## 1. Introduction

Macrophage migration inhibitory factor (MIF) is a pleiotropic cytokine [1] that is also a mediator of innate and adaptive immune responses [2]. In humans, it is present within the cytosol of cells as a pre-formed protein. Several MIF effects are mediated via an autocrine/paracrine signaling pathway, leading to the activation of ERK1/ERK2 MAP kinases, the triggering of a downstream pro-inflammatory gene expression, the production of matrix metalloproteases, the upregulation of TLR4 expression, the suppression of p53 activity, the regulation of the anti-inflammatory and immunosuppressive effects of glucocorticoids, and the regulation of cell cycling [3,4].

Genes encoding MIF proteins have been found in prokaryotes, vertebrates, invertebrates, and plants [5]. In the Ascidian *Ciona robusta*, two macrophage migration inhibitory factor (*Mif*) genes have been identified: *Mif-1* and *Mif-2* [6]. *C. robusta*, the closest living relative of vertebrates, has become a model in various fields of biology, serving as a particularly powerful model for studying innate immunity [7,8,9,10]. In *C. robusta*, challenges with pathogen-associated molecular patterns (PAMPs), such as Gram-negative lipopolysaccharide (LPS), induces inflammatory-like reactions in the pharynx (hematopoietic organ) [11] and the expression of several innate immune genes in granulocyte hemocytes, such as cytokines, galectins, mannose binding lectin (*Mbl*) and pentraxin (*Ptx-like*) [11,12]. Furthermore, transcriptome data analyses suggest a close interplay between Mif cytokines and signaling pathway components of the nuclear factor kappa-light-chain-enhancer of activated B cells (Nf-κB) [13]. Part of the explanation of the potential role of MIF cytokines in inflammation in humans was given by the identification of the interaction of JAB1/CSN5 with the tumor suppressor p53 [14], offering an intriguing molecular connection between MIF-mediated signaling, the COP9 (Constitutive photomorphogenesis 9) signalosome (CSN) complex, and MIF-modulated cell inflammatory and proliferation effects. Indeed, JAB1/CSN5 is part of a fine-tuning of the activation and switching-off of these MIF-driven signaling pathways, such as the ERK1/2 MAPK and Nf-kB pathways [15].

CSN5 is part of a conserved multiprotein complex belonging to the deubiquitylating enzymes (DUBs) [16], which is mainly known for its role in the control of the proteolysis and deadenylation processes. It exerts its main function together with other accessory proteins, including cullins, the F-box/LRR-repeat protein (FBXL), Ring-box proteins (RBX), and the cullin-associated Nedd8-dissociated protein 1 (CAND-1) [17]. Moreover, due to its position in a central cellular pathway connected to cell responses such as cell cycle, proliferation, and signaling, CSN is involved in the regulation of several human diseases and processes linked to the innate and adaptive immune response, wound healing, tissue maintenance, and in the development of immune cells [18]. Emerging evidence also suggests that CSN is involved in inflammation. This is both due to its role in controlling cullin-RING ubiquitin ligases (CRLs), thus regulating key components of inflammatory pathways such as NF-kB, and complex-independent interactions of subunits, such as CSN5 with inflammatory proteins [17,19]. One of the first pieces of evidence of a functional link between the CSN and NF-κB signaling was identified by Schweitzer et al. [20]. They found that CSN controls the activity of CRL-β-TrCP that ubiquitinates the NF-κB inhibitor IκBα. Interestingly, CSN is also associated with USP15, thereby promoting IκBα deubiquitination and allowing precise regulation of NF-κB signaling [21]. A regulative impact of CSN on NF-κB signaling was also shown in the HEK293 fibroblast cell line, where CSN was observed to interact with the IκBα phosphorylating IKK complex and to promote its activity [21]. Although genes known to be involved in immune response, such as *TLR*, *NF-kB*, and *MIF*, are expressed in *C. robusta*, the wide-ranging nature and temporal dynamics of immune signaling in *C. robusta* during LPS exposure in vivo remain unclear.

In the present study, using a combined approach with an in-vivo LPS exposure strategy, next-generation sequencing (NGS), qRT-PCR analysis, bioinformatics, and in-silico analyses, we suggest that in *C. robusta*, Mif cytokines and the CSN signaling platform are involved in the regulation of Nf-kB in the innate immune response during LPS-mediated inflammation.

## 2. Results

### 2.1. Transcriptomic Analysis of C. robusta Pharynx 

To highlight potential molecules involved in the inflammatory response under LPS exposure, we performed a transcriptomic analysis of *C. robusta’s* immunocompetent organ under both physiological and challenged conditions (LPS treatment at 4 h) [13]. NGS analysis identified both protein coding and non-coding RNAs (microRNAs). (All transcriptome expression data are available in the Supplementary Material, File S1, sheet 1-transcript all, sheet 8-miRNAs in NGS).

Although NGS differential expression (DE) analysis and Gene Ontology (GO) functional annotation showed that some genes linked to immune system processes were slightly modulated at 4 h of exposition to the LPS agent [13], genes belonging to the Cop9 signaling network were not modulated at this time point. Table 1 shows immunogens that are deregulated at 4 h of LPS induction. Between these genes just few genes of the Mif-Cop9-Nf-kB network were modulated (Mif-1, Nf-kB, If5-like, Hsp70). Table 2 shows all genes belonging to the Mif-Cop9-Nf-kB network, both deregulated and not modulated.

### 2.2. Analyses of the Expression of the Mif-Csn-Nf-κB Signaling Network Genes under LPS Exposure

**Figure 1:**
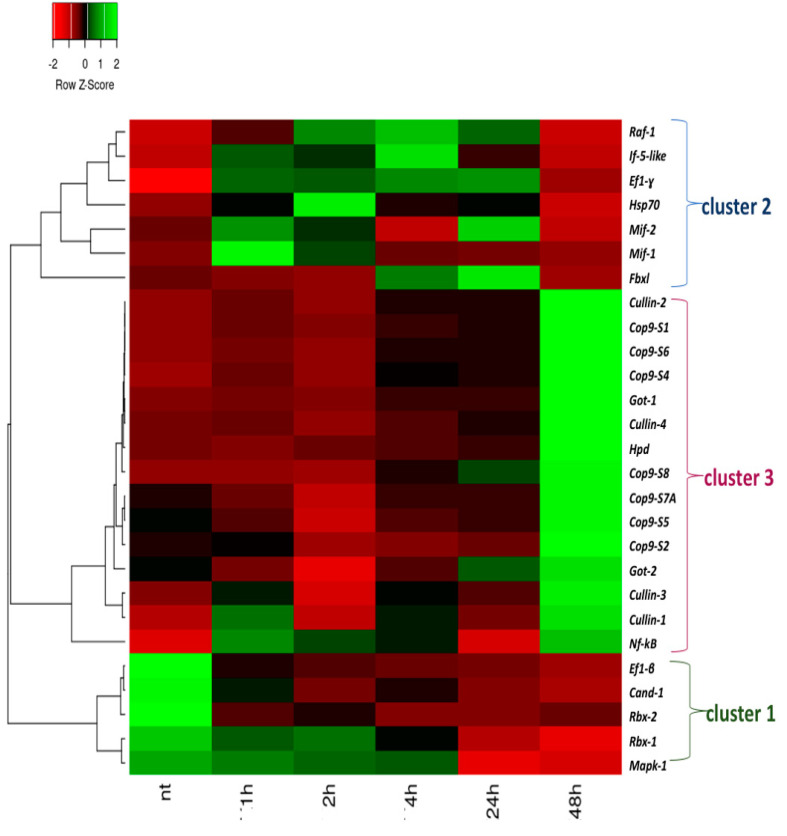
Heatmap based on qRT-PCR analysis of the differentially expressed *Mif-Csn-Nf-kB* signaling network genes under LPS exposure. The figure shows LPS-treated (1 h, 2 h, 4 h, 24 h, 48 h) and untreated samples (nt). Time course of gene expression in the pharynx of *C. robusta* exposed to LPS compared with the gene expression in untreated ascidians. Values are represented according to the *z*-score, a measure that describes a value’s relationship to the mean of a group of values, which is measured in terms of standard deviations from the mean. The heatmap was generated using the complete linkage as a clustering algorithm and the Pearson correlation as a distance measurement method. Clusters 1, 2 and 3 are showed in Figure 1, in accordance with expression values at different time-points.

Analyses of the time-course expression of *Mif-Cop9-Nf-κB* network genes in pharynx inflammatory response induced by LPS in *C. robusta* were performed at time points from 0 to 48 h post LPS challenge by qRT-PCR (Figure 1). 

The heatmap shows that a large portion of the transcripts were significantly modulated in response to LPS during the 48 h period of LPS exposure. Based on the expression patterns of the transcripts, three major clusters were highlighted: a first cluster of genes is activated very early after the LPS challenge, which is cluster 1 (cluster 1). It includes *Ef1-ß, Cand-1, Rbx-2, Rbx-1,* and *Mapk-1*, which resulted down-regulation in the intermediate and late phases of the inflammation process. The second cluster includes *Raf-1, If5-like, Ef-1γ, Hsp70, Mif-1, Mif-2,* and *Fbxl*, which were modulated between 1h and 24h after LPS exposure (cluster 2), and are activated in the progression of the inflammation process; the third includes genes involved in the *Csn* complex (Csn subunits and cullin family) and some immune, inflammatory, and metabolism genes such as *Got-1*, *Got-2* and *Hpd*, which were highly expressed at 48 h, along with *Nf-kB* (cluster 3).

### 2.3. Phylogenetic Analysis of CSN and In-Silico Analyses of Csn Interactors

**Figure 2:**
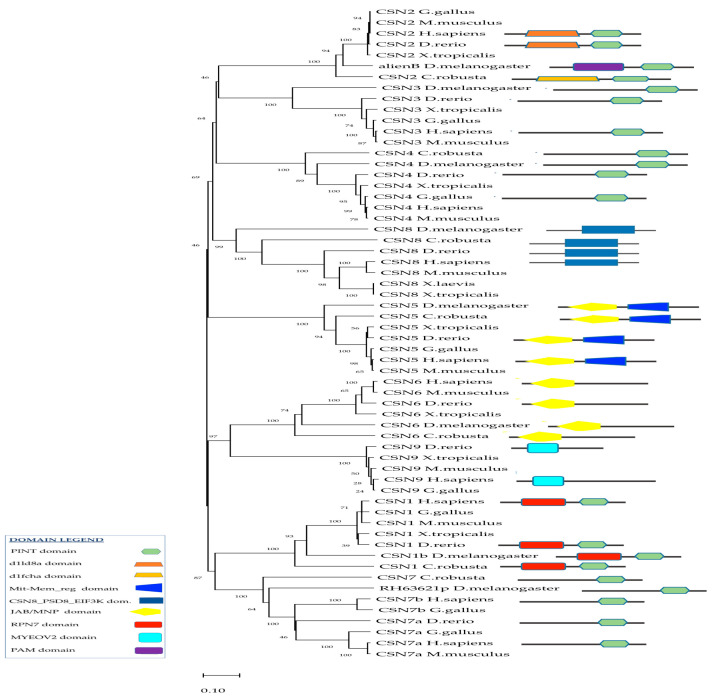
Phylogenetic tree and domain structure of CSN proteins. The tree was constructed by the neighbor-joining method and bootstrap analysis by MEGA 11. The bootstrap value indicates the number of particular node occurrences per 1000 trees generated by bootstrapping the sequences, expressed as a percentage. The evolutionary distances were computed using the Poisson correction method and are in the units of the number of amino acid substitutions per site.

In humans, the C-terminal regions of CSN1, CSN2, CSN3, CSN4, and CSN7 contain a conserved PINT domain (Figure 2). It mediates and stabilizes protein-protein interactions within the complexes, supporting intra-complex interactions as well as the recruitment of additional ligands. The N-terminal region of CSN5 and CSN6 contained a JAB/MPN domain (Figure 2). In *C. robusta*, the Cop9 signaling complex consists of different subunits from SCN1 to SCN8, whereas CSN3 is not present. In-silico analysis using the Simple Modular Architecture Research Tool (SMART) (http://smart.embl-heidelberg.de/ accessed on 30 August 2022) and GenBank analyses through the Basic Local Alignment Search Tool (BLAST) (https://blast.ncbi.nlm.nih.gov accessed on 30 August 2022) revealed that in *C. robusta*, the domain structures of the Csn component were consistent with their orthologs in humans. In particular, Csn1, Csn2, Csn4, Csn7-like, and Csn8-like subunits show a highly conserved PINT domain, while Csn5 and Csn6 have a JAB/MPN domain (Figure 2, Table 3). Moreover, in human and *C. robusta* CSN1 has the regulatory subunit RPN7 (known as the non-ATPase regulatory subunit 6 in higher eukaryotes) of the 26S proteasome, associated with the PCI/PINT domain. CSN2 subunits have specific domains associated with the PINT domain, which are respectively the d1ld8a domain for humans and d1fcha for *C. robusta.* CSN6 subunits have a Mit-Mem_reg domain, which is found at the C-terminus of many regulatory proteins, including the yeast proteasomal subunit Rpn11 and eukaryotic initiation factor 3 subunit F (eIF3f). CSN8 subunits have a CSN8/PSMD8/EIF3K domain, which are conserved from fungi, and from plants to humans. In the CSN2 of *D. melanogaster*, a PAM domain is present, that is an associated module with an all-alpha-helix fold to PCI/ PINT. In *H. sapiens* and the *D. rerio* CSN9 subunit, a MYOV2 domain is present, which is a Phe/Asp-rich domain necessary for its incorporation into the CSN complex.

Phylogenetic analyses of vertebrate and invertebrate CSN proteins supported the idea of a conserved evolution from a common ancestral gene among invertebrates, protochordates, and vertebrates. The same homologous CSN protein subunits in different species were clustered in the same branch (Figure 2). 

In humans, protein interactors such as cullins, RBX proteins, CAND-1, and FBXL are involved in Csn functions. Cullins are a very conserved protein family, each forming part of a multi-subunit ubiquitin complex. Their N-terminal region is more variable and is used to interact with specific adaptor proteins. They also have a neddylation domain. RBX-1 and RBX-2 are part of a cullin-RING ubiquitin ligase complex, in which the RING domain constitutes the catalytic core. The C-terminal region allows them to bind the RING protein. Meanwhile, the *H. sapiens* CAND-1 has only low complexity regions (heat repeats).

In *C. robusta*, Cul-1, Cul-2, Cul-3, and Cul-4 show a highly conserved cullin domain (File S1, Supplementary Material) of cullin domains in *H. sapiens, D. Melanogaster*, and *C. robusta*), and all of them have both the cullin and neddylation domains, with the exception of Cul-3, which has no neddylation domain. They have a percentage of amino acid sequence identity with humans ranging from 62.7% to 72.9%. *C. robusta* Rbx-1 and Rbx-2 show a sequence identity greater than 82% compared with *H. sapiens* RBX proteins (https://blast.ncbi.nlm.nih.gov/ accessed on 30 August 2022). *C. robusta* Cand-1 and Fbxl have an aminoacidic sequence identity, respectively, of 56.3% and 24.6% with human orthologs. 

### 2.4. PPI analysis of C. robusta and H. sapiens Mif-Cop9-Nf-kB Signalosome Network 

**Figure 3:**
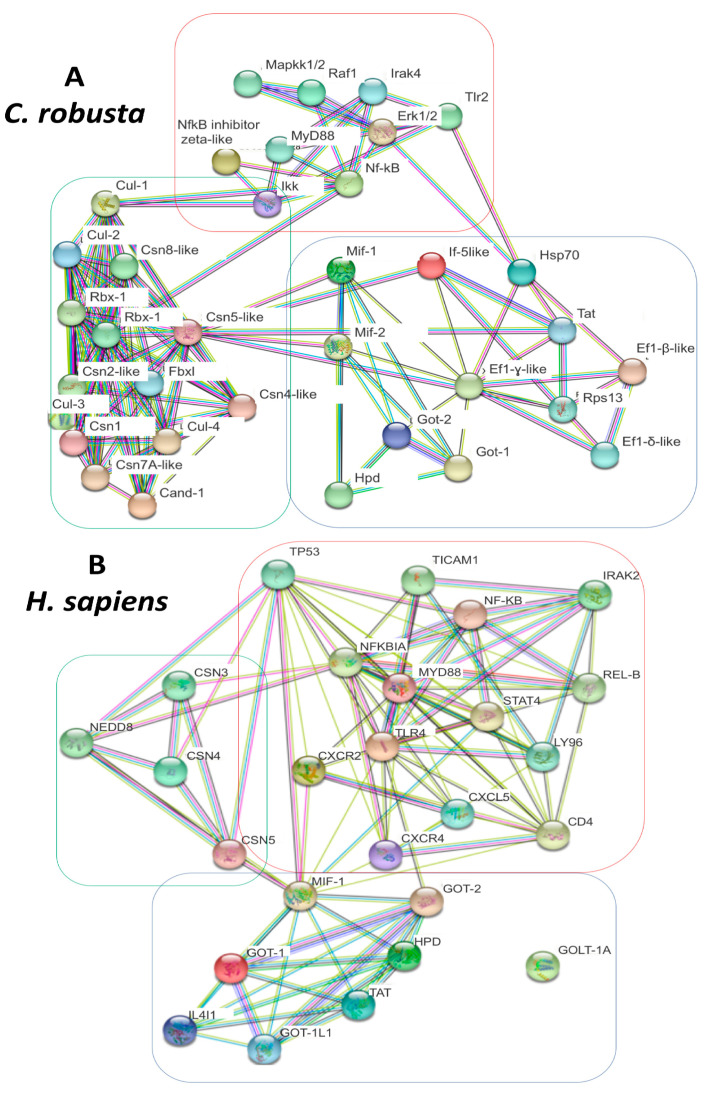
*C. robusta* (**A**) and *H. sapiens* (**B**) STRING networks are shown. Proteins are shown as network nodes; interactions are the edges. More edges are present between two nodes, and more pieces of evidence connect the two interacting proteins. Each node color represents a different protein of the network; edge colors represent different kind of direct and indirect interactions that are the following: known interactions (light blue and pink), predicted interactions (green, red and blue) and others (text-mining, co-expression and protein homology). Three different protein clusters are evidenced both in *C. robusta* and in *H. sapiens*, and they are colored green, light blue and red.

A functional analysis of STRING-protein–protein interaction (PPI) networks (string-db.org) was used to predict the interactions among proteins linked to the MIF-CSN-NF-kB signaling network. The analysis was performed in both *C. robusta* and *H. sapiens* species to provide evidence of differences and similarities between the two pathways (Figure 3A,B). PPI networks revealed an interplay between MIF and the CSN complex in both *C. robusta* and *H. sapiens*. The main protein involved in this interaction is subunit 5 of the CSN signaling complex (CSN5), which directly interacts with MIF proteins. Indeed, focusing on the *C. robusta* PPI network, Csn5 is the main hub node of different interactions with molecules that are involved in many steps of the inflammation signaling cascade, for example Mif proteins. Moreover, it interacts with proteins of the translation machinery, such as If5, or metabolic processes such as Got proteins. It is also involved in different biological processes including cellular metabolic processes (GO:0044267, FDR 8.67 × 10^−12^ organonitrogen compound GO:1901564, FDR 2.38 × 10^−10^ Protein deneddylation GO:0000338, FDR 1.34 × 10^−9^ protein modification by small protein conjugation GO:0070647, FDR 1.28 × 10^−7^ cellular protein modification processes GO:0006464, FDR 1.28 × 10^−7^ primary metabolic process GO:0044238, FDR 1.40 × 10^−7^ cellular metabolic process GO:0044237, FDR 2.17 × 10^−6^ (File S2, and Supplementary Material, File S1 sheet 6 to sheet 14). There is also a small group of translational machinery proteins interacting with Mif-2: eukaryotic translation initiation factor 5A-like (If-5-like), elongation factor 1-beta/delta/gamma-like isoforms (Ef1-β/δ/γ-like), and ribosomal protein S13 (Rps13). STRING analysis also evidenced proteins belonging to the Tlr2-Nf-kB and MyD88 networks. In addition to Csn subunit components, other proteins belonging to the Csn network were found, such as the cullin family, Fbxl, Rbx-1, Rbx-2, and Cand-1. The third class of proteins evidenced by STRING are those related to the metabolism of amino acids, such as aspartate aminotransferase cytoplasmic-like (Got-1), aspartate aminotransferase mitochondrial (Got-2), and 4-hydroxyphenylpyruvate dioxygenase (Hpd). STRING analysis of the *H. sapiens* network also evidenced inflammation network proteins, such as NF-KB, IRAK2, TLR4, and REL-B, proteins of aminoacidic metabolism, and the CSN complex (CSN5, CSN4, CSN3, and NEDD8), as well as chemokines such as CXCL5, CXCR4, and CXCR2. 

### 2.5. Post-Transcriptional Regulation of Mif-Cop9 Signalosome-Nf-kB Network

We performed miRNA-target interaction prediction through the MiRNATIP algorithm to find evidence for potential interactions between the *Mif-Csn-Nf-kB* axis and miRNAs, previously identified with NGS analysis. Algorithm results were filtered for energy interaction values < 12 kcal/mol to reinforce the results of the predictions. Both conserved and species-specific miRNAs were predicted (Supplementary Material, File S1, sheet 3-miRNA-target prediction, sheet 4-prediction filtered for energy value). 

Table 4 and Table 5 show the list of conserved and species-specific miRNA that the MiRNATIP algorithm predicted to interact, respectively, with Csn subunits, cullin family members, *Rbx-1* and *Rbx-2*; *Cand-1*, *Mif-1*, *Mif-2*, and *Nf-kB* target transcripts. The majority of conserved miRNAs are involved in the inflammation process (Table 4). For species-specific miRNAs (Table 5), there is no information in the literature about any involvement with the inflammation process, with the exception of cin-miR-4189 [22].

Only *Mif-1* and *Mif-2* targets have been shown to interact only with species-specific miRNAs; for them, the MiRNATIP algorithm did not demonstrate any conserved interacting miRNAs; the other targets have both conserved and species-specific interacting miRNAs (a detailed list of miRNA-target predictions is in the Supplementary Material, File S1, sheet3-miRNA-target prediction, and sheet4-prediction filtered NGS, and a detailed list of species-specific miRNAs is in Supplementary Material, File S2, specie-specific miRNAs).

### 2.6. Network Reconstruction of Post-Transcriptional Regulation of Mif-Cop9 Signalosome-Nf-kB Network and Target Genes Interplay

**Figure 4:**
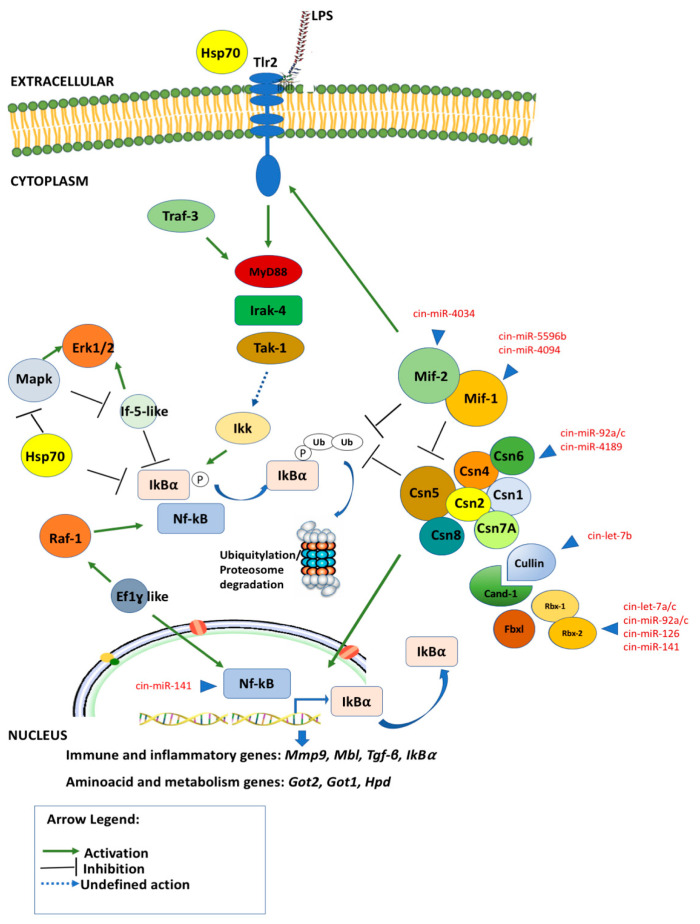
The Mif-Csn interplay in the regulation of Nf-kB in the innate immune response during LPS-mediated inflammation and their post-transcriptional regulation through miRNA action. All the molecules shown in the figure have been detected in the *C. robusta* transcriptome.

A novel schematic model representing the putative interplay between the Mif, Csn, and Nf-κB pathway components identified by a combined approach using NGS, qRT-PCR, STRING and miRNA targets analysis in *C. robusta* is shown in Figure 4.

External immunological stimuli, such as LPS, can activate a signaling cascade that is first mediated by the Tlr2 membrane signaling receptor and then activates cytosol molecules involved in inflammatory processes such as Irak4, MyD88, Ikk, Nf-kB, Erk1/2. 

The data produced by NGS, qRT-PCR and STRING interactions provide evidence of immune genes belonging to three different phases of the inflammation process, which are an initialization, a progression and finally a resolution of the inflammation process. These phases are well characterized by three different clusters of genes evidenced by qRT-PCR analysis. Indeed, the first cluster includes a group of genes (cluster 1 of qRT-PCR, Figure 1) that can be identified as an entry point of the regulatory cascade, that once triggered by the inflammation stimulus (LPS), activate the transcription of the second group of genes (cluster 2 of qRT-PCR, Figure 1), which regulate the expression of Nf-kB factor from 2 h to 24/48 h, driving the inflammatory response. The third group of genes found activated at 48 h (cluster 3 of qRT-PCR, Figure 1) lead the inflammatory response to the end, restoring the homeostasis.

After the activation of Tlr2 receptor and its downstream signaling partners (MyD88, Traf3 and Irak4), there are some proteins such as the transcription factor Ef1-ß, and other proteins such as Cand-1, Rbx-2, Rbx-1; these help the Cop9 signaling complex in its multifunctional role, are present in the very early stage of the inflammation process, and assist the transcription of the second group of genes, such as *Raf-1, If5-like, Ef-1γ, Hsp70, Mif-1, Mif-2, Fbxl*. Indeed, this second cluster of genes is modulated between 1 and 24 h after LPS exposure (cluster 2), being probably activated during the progression of the inflammation process.

Nf-kB is a protein complex that controls the transcription of DNA, cytokine production and cell survival, and it is one of the main actors in the inflammation processes. It is mainly regulated by IκBα which represents its major negative Nf-κB feedback loop. 

Furthermore, Mif-1 and Mif-2 proteins also interact together with the Csn complex in Nf-kB regulation; both intervening in facilitating the Ikk complex activation in the cytoplasm, and thus contributing to the down-regulation of persistent Nf-kB signaling via stabilization of re-synthesized IκBα by deubiquitylation, and through regulating the Csn5 function itself by inhibiting the Cop9 subunit. Hsp70 has also shown to be present in the cytosol and intervening with anti-inflammatory effects involved in Nf-kB regulation during the progression of inflammation. 

Nf-kB phosphorylation is regulated by other signaling cascades, such as that involving Raf-1/Erk1/2 signaling. Raf-1 leads to Nf-kB phosphorylation, which leads to the enhancement of ILs gene expression, prolonging Nf-κB activity, and increasing the transcription rate from the downstream *IL* genes. Thus, Nf-kB cannot be assembled with IκB, preventing the inactivation and nuclear export of Nf-κB to the cytoplasm. Raf-1 also interacts with translation machinery during signal transduction, such as Ef1, playing a role in regulating the duration of NF-kB nuclear occupancy. Another molecule involved in Raf-1/Erk1/2 signaling is eIf5A, which causes the inhibition of phosphorylation and Erk-MapK and Nf-κB activity due to the increase of hypusine, which fuels biosynthesis and is increased during inflammation.

During the final steps of the inflammation process and the initial restoration of homeostatic condition, a third cluster of genes is expressed. It includes genes involved in the *Csn* complex (Csn subunits and cullin family) and some immune, inflammatory, and metabolism genes, such as *Got-1*, *Got-2* and *Hpd*, which were highly expressed at 48 h along with *Nf-kB.* This modulation of proteins related to the metabolism of amino acids, such as Got-1, Got-2, Hpd, is probably linked to the increase in cytokines production leading to metabolic changes caused by the need to modify protein and amino acid requirements. During immunological stress, amino acids are redistributed away from protein production (growth, lactation, etc.) and towards tissues involved in inflammation and immune response. They are used for the synthesis of inflammatory and immune proteins, to support immune cell proliferation, and for the synthesis of other compounds essential for body defense functions.

Finally, Nf-kB expression is both at the beginning and during the late inflammation time point, probably with a dual role both in the initiation and the resolution of the inflammation process. This suggests a biphasic activation of the inflammatory response upon LPS exposure, with a first wave of activation at early phases after LPS challenge, and a second wave of activation at 48 h. 

In the immunity response, the LPS stimuli starts a molecular mechanism that leads to transcriptional ‘on’ and ‘off’ switches on many immune genes which are coordinately and temporally regulated at many levels. This sequence of events is important in coordinating all the different steps of inflammation evolution. Indeed, the Mif-Cop9-Nf-kB signaling network is finely regulated by non-coding molecules such as miRNAs. They interact with different target genes involved in initiation, progression and resolution of the inflammation process. Some of these miRNAs are known to be involved in the inflammation process in *H. sapiens*, such as the cin-let-7 and cin-miR-92 families, which interact with Cullin family members and Cop9 signaling complex proteins. Other target genes such as Mif-1 and Mif-2 are targeted by species-specific miRNAs that modulate their expression by a specific seed-sequence interaction, such as cin-miR-4034, cin-miR-5596b, and cin-miR 4094. The Nf-kB expression is also regulated by the miRNA interaction as shown in Figure 4. 

## 3. Discussion

The innate immune signaling pathway activated by LPS in *C. robusta* is evolutionarily conserved, and previous findings have shown that the Mif signaling pathway is activated after LPS stimuli as a first-line defense mechanism against pathogens, leading to the activation of Nf-kB signaling [13,29,30]. Recently, in *H. sapiens* a Cop 9 signaling complex was also shown to be connected with NF-kB signaling [31]. 

In this study, to highlight a new PPI network linked to the Mif and Nf-kB signal pathways, a combined approach using NGS, qRT-PCR, and STRING analysis was utilized. STRING analysis has shown three different clusters of Mif-Csn-Nf-κB signaling network genes in *C. robusta* which were identified by an NGS strategy (the tree clusters showed are highlighted in STRING Figure 3 as red, green and light blue).

The first cluster involved molecules linked to the Nf-kB signaling cascade, ranging from the Tlr2 membrane signaling receptor to cytosol molecules such as Irak4, MyD88, Ikk, Nf-kB, Erk1/2, and Hsp70 (red cluster highlighted in STRING, Figure 3). In *H. sapiens,* NF-κB is the major regulator of pro-inflammatory gene expression after LPS-stimuli in macrophages [32,33,34]. Recently, heat shock proteins (HSPs) were found to be associated with anti-inflammatory effects as intracellular proteins, inhibiting the activation of NF-kB [35,36].

HSPs are a group of well-conserved stress proteins that maintain protein homeostasis by counteracting protein denaturation, preventing protein misfolding and assisting in assembly. Interestingly, HSP70 can also play an opposite role when it acts as an extracellular protein by interacting with TLR2/4 and activating the NF-kB inflammatory pathway [34,37,38,39].

In *C. robusta*, as in humans, a STRING cluster including different subunits of the Csn complex, together with cullin protein family members, FBXL, CAND-1, and RBX proteins, was also identified (green cluster highlighted in STRING, Figure 3). The main protein involved in PPI, evidenced by STRING analysis, was subunit 5 of the Cop9 signaling complex which directly interacts with the Mif-1 and Mif-2 proteins in *C. robusta* and MIF in humans. There are many activities in which the Cop9 signaling complex is involved in vertebrates, and the most well-known is the regulation of protein stability through proteasome complex involvement and protein deneddylation. Other functions of CSN are transcription activation of interacting targets, and finally, the regulation of subcellular localization of downstream proteins [40].

Considering the high degree of conservation among the different subunits of the Cop9 complex in invertebrates and vertebrates, as confirmed by phylogenetic analysis, we hypothesize that the *C. robusta* Cop9 signaling complex could also be involved in the same functions identified in vertebrates [31,40,41]. Protein phosphorylation is perhaps the best-known function of the Cop9 signaling complex. Indeed, CSN5 was shown to regulate the activation of the IkBα−NF-kB complex through the phosphorylation/deubiquitilation of IkBα. Furthermore, the recruitment and activation of the IKK complex resulted in the phosphorylation, ubiquitylation, and proteasomal degradation of the NF-κB inhibitor IκBα, thus resulting in the release of active NF-κB. IκBα is one of the NF-kB target genes, which represents a major negative NF-κB feedback loop. On the one hand, the CSN might intervene in facilitating IKK complex activation in the cytoplasm, and it could contribute to the down-regulation of persistent NF-kB signaling via stabilization of re-synthesized IκBα by deubiquitylation [17,21]. 

The last cluster shown by STRING analysis in *C. robusta* involves Mif-1 and Mif-2, certain proteins related to the metabolism of amino acids, such as Got-1, Got-2, Hpd, and factors linked to the translation process such as If-5-like, Rps13, and Ef-1- β/δ *γ* isoforms (light blue cluster highlighted in STRING, Figure 3).

*Got-1, Got-2*, and *Hpd* genes are involved in metabolic changes associated with inflammatory processes and infectious diseases when it is needed to modify protein and amino acid requirements [42]. This is thought to be the consequence of an increase in the production of cytokines, such as interleukins acting to alter protein metabolism. During immunological stress, amino acids are redistributed away from protein production (growth, lactation, etc.) and towards tissues involved in inflammation and immune response. They are used for the synthesis of inflammatory and immune proteins, to support immune cell proliferation, and for the synthesis of other compounds important for body defense functions. Therefore, the stimulation of the immune system disturbs normal body processes, and in turn is able to induce specific amino acid requirements [42]. 

RAF-1, identified in the first cluster of the STRING analysis of *C. robusta*, is involved in controlling the adaptive immune responses in humans [43]. It leads to NF-kB phosphorylation and the subsequent acetylation of p65 subunits, leading to the enhancement of ILs gene expression, prolonging NF-κB activity, and increasing the transcription rate from the downstream *IL* genes. Thus, NF-kB cannot be assembled with IκB, preventing the inactivation and nuclear export of NF-κB to the cytoplasm [44]. The human kinase RAF-1 also interacts with translation machinery during signal transduction, as demonstrated by Migliaccio et al. [45]. Indeed, a particular emphasis is given to an intriguing non-canonical role that EF-1 can play in the signaling of RAF-1-ERK-1/2. Moreover, the deregulation of IF-5 expression levels can modulate the ERK signaling cascade, thus participating in the RAF-1-ERK-1/2 cascade. IF-1 also plays a role intervening in regulating the duration of NF-kB nuclear occupancy. Indeed, in humans, eEF-1A1 forms complexes with STAT3 and PKCδ, which are crucial for STAT3 phosphorylation and for NF-κB/STAT3-enhanced interleukin-6 expression [46]. Nf-kB activity is also regulated by eIF5A. It is an abundant, constitutively expressed protein, and it is the only known protein to contain the unique amino acid hypusine which fuels biosynthesis and is increased during inflammation [47]. The reduction of hypusine-modified eIF5A levels caused the inhibition of phosphorylation and ERK-MAPK and NF-κB activity [48].

The time-course expression of the *Mif-Csn-Nf-κB* network genes in the pharynx inflammatory response induced by LPS from 0 to 48 h time points evidenced two upregulated groups and a third one which was downregulated. The first group of upregulated genes included *Raf-1*, *If5-like*, *Ef1-γ*, *Hsp70*, *Mif-1*, *Mif-2*, and *Fbxl*, which were modulated between 1 and 24 h after LPS exposure; the second included genes involved in the *Csn* complex (Csn subunits and cullin family) and some immune, inflammatory and metabolism genes such as *Got-1*, *Got-2* and *Hpd*, which were highly expressed at 48 h together with *Nf-kB*; the third group included *Ef1-ß*, *Cand-1*, *Rbx-2*, *Rbx-1*, and *Mapk-1*, which were down-regulated between 1 and 48 h after LPS exposure. In accordance with previous results [13], our findings suggest an intriguing possibility of the biphasic activation of the inflammatory response upon LPS exposure, with the first wave of pro-inflammatory activation from 0 to 24 h and a second wave of anti-inflammatory action later at 48 h. 

Interestingly, these data were in agreement with those obtained by STRING analysis, suggesting a highly conserved functional link between the molecules highlighted in the three clusters of the STRING analysis and closely related to the inflammatory phenomenon induced by LPS in *C. robusta*.

As we know, recent evidence suggests that gene expression may be regulated, at least in part, at the post-transcriptional level by factors inducing the extremely rapid degradation of messenger RNAs. This is one of the various molecular mechanisms governing the activation or inhibition of gene expression. Non-coding molecules are small RNA molecules that effectively play this role in many physiological and pathological states. Predictions of miRNA-target interactions through the MiRNATIP algorithm [49] were performed to investigate which miRNAs might be involved in the regulation of the Mif-Csn-Nf-kB pathway in *C. robusta*. Different and conserved miRNAs (cin-let7a/b/c, cin-miR-92a/c, cin-miR-141, and cin-miR-126), and one species-specific miRNA (cin-miR-4189) identified in the *C. robusta* transcriptome were shown to be involved in the post-transcriptional regulation of these molecules [50]. 

Let-7 family members are one of the most studied conserved miRNA families linked to inflammation. Indeed, human let-7e and let-7b are known to promote NF-κB activation and translocation to the nucleus by inhibiting its target gene (Iκβ) expression and subsequently increasing the expression of pro-inflammatory and adhesion molecules [24,51]. On the contrary, other family members, such as let-7c, inhibit the inflammatory response in LPS-treated cells [23]. Furthermore, other conserved miRNAs are involved in the regulation of inflammation process, acting on different targets of the Nf-kB pathway. One example is miR92b, which attenuated inflammatory response and autophagy by down-regulating TRAF3 and suppressing the MKK3-p38 pathway in acute pancreatitis inflammatory disease [52]. Interestingly, *Mif-1* and *Mif-2* genes do not interact with conserved miRNAs, but they have been predicted to bind only to species-specific miRNAs (Figure 4). Recently, in *C. robusta* [22,23,24,50,51,52], several species-specific miRNAs were identified as being involved in post-transcriptional regulation control of immune genes. In ascidian *C. robusta*, transcriptome analysis showed that Mif, Csn, and Nf-κB pathway components are similar to those found in humans, in innate immunity response. The RT-PCR analysis shows that those molecules can be induced by LPS; Phylogenetic, miRNA-target prediction and STRING analyses show that in ascidian *C. robusta*, sophisticated signaling and behaviors based on dynamic feedback regulated interactions among a number of components (genes, coding and non-coding transcripts) can interact in a highly conserved way in the course of evolution. All these results let us hypothesize a conserved complex system in which different molecules extensively crosstalk at many levels in the innate immune response during the LPS inflammation process in *C. robusta*.

## 4. Materials and Methods

### 4.1. Tunicates and LPS Injection

*C. robusta* specimens, formerly classified as *C. intestinalis* [53,54,55,56,57], were collected from Sciacca Harbor (Sicily, Italy) and acclimatized at 15 °C in tanks supplied with flow-through oxygenated seawater. One hundred microliters of LPS solution (*Escherichia coli* 055:B5, LPS, Sigma-Aldrich, St. Louis, Missouri (Mo), United States (USA); 12 mM CaCl_2_, 11 mM KCl, 26 mM MgCl_2_, 43 mM Tris HCl, 0.4 M NaCl, pH 8.0) were injected into the tunic matrix surrounding the pharynx wall (median body region) at a final LPS concentration of 100 μg. *C. robusta* specimens that were not exposed to LPS (naïve) were used as controls.

### 4.2. Next-Generation Sequencing

The RNA purity and quality of total RNA extracted from the pharynx of *C. robusta* was assessed for 3 naïve replicates (*n* = 3) and 3 replicates (*n* = 3) that were exposed to LPS for 4 h [13]. RNA sequencing (RNA-Seq) was performed by BMR Genomics (Padua, Italy) on an Illumina platform in a single-end format 75 bp (1X75 bp) containing ~40 million of 10% reads/sample [13]. In the following, we report the pipeline used for the in-silico analysis provided by the BMR genomics (Padua, Italy). First, the read quality check was performed using the FastQC (v.0.11.7) tool (FastQC2018 https://www.bioinformatics.babraham.ac.uk/projects/fastqc, accessed on 1 June 2018) to remove low-quality or corrupted reads. Then the remaining reads with the *C. robusta* reference Genome assembly KH (GCA_000224145.1) were aligned using the Hisat2 (v.2.0.5) tool [58], providing both coding and non-coding genes annotations. At this point, the featureCounts (v. 1.5.2) algorithm [59] was used to create the row count matrix. Finally, a differential expression analysis between treated (4h LPS induction) and untreated coding genes using the EdgeR (v.3.16.0) software EdgeR2010 [60] was performed, which estimated the negative binomial variance parameter globally across all genes.

### 4.3. qRT-PCR

The differential expression of Mif-Csn-Nf-kB axis LPS-responsive genes was studied by qRT-PCR using the SYBR-Green method and the specific sets of primers listed in File S1. qRT-PCR analysis was performed using an Applied Biosystems 7500 Real-time PCR system [61,62]. Differential expression was determined in a 25 μL PCR mixture containing 2 μL of cDNA converted from 250 ng of total RNA, 300 nM primer (forward and reverse), and 12.5 μL of Power SYBR-Green PCR MasterMix (Applied Biosystems, Waltham, MA, USA).

Amplification specificity was tested using a real-time PCR melting analysis. To obtain sample quantification, the 2^−ΔΔCt^ method was used, and the relative changes in gene expression were analyzed as described in the Applied Biosystems Use Bulletin N. 2 (P/N 4303859).

The transcript levels from different tissues were normalized to that of actin to compensate for variations in the amount of RNA input. Relative expression was determined by dividing the normalized value of the target gene in each tissue by the normalized value obtained from the untreated tissue. To examine the time course of the response, 4 LPS-treated ascidian replicates (*n* = 4) were examined at incremental post-inoculation time points (1, 2, 4, 8, 12, 24, and 48 h). Four untreated (naïve) ascidian replicates (*n* = 4) were used as controls. A heatmap was generated to visualize the results indicating the genes differentially expressed between the exposed samples and controls (LPS exposure times were 1 h, 2 h, 4 h, 24 h, and 48 h). The Minitab 17 statistical software was used for the qRT-PCR data analysis. Statistical differences were estimated by a one-way ANOVA, and the significance of differences among groups was determined by Tukey’s *t*-test. The level of significance was set at a *p*-value ≤ 0.05. The data are presented as the means ± SD (*n* = 4).

The heatmap was produced using a heatmapping tool (https://www.heatmapper.ca accessed on 1 June 2022). Complete linkage clustering was applied, and Pearson correlation was used as the method of distance measurement. Additionally, a *z*-score was calculated, a measure that describes a value’s relationship to the mean of a group of values. The *z*-score was measured in terms of standard deviations from the mean [63].

### 4.4. Phylogenetic Analyses of Csn Complex and In-Silico Analysis of Csn Partners

The phylogenetic tree was obtained using the neighbor-joining method [64]. The tree is drawn to scale, with branch lengths in the same units as those of the evolutionary distances used to infer the phylogenetic tree. The evolutionary distances were computed using the Poisson correction method [65] and are in the units of the number of amino acid substitutions per site. Evolutionary analyses were conducted in MEGA 11 [66].

The accession numbers are listed in File S2.

Analysis using the BLAST tool (https://blast.ncbi.nlm.nih.gov/, accessed on 30 August 2022) was performed, starting from *C. robusta* protein sequences, by using the Uniprot database (https://www.uniprot.org/blast, accessed on 30 August 2022). *C. robusta* sequences were blasted against *H. sapiens* using the BLASTP program, with an E-threshold of 1 × 10^−10^ and the autoblosum62 matrix to perform sequence analysis. 

### 4.5. STRING Analysis of C. robusta and H. sapiens Mif-Cop9 Interaction Network

The STRING database (https://cn.string-db.org, accessed on 6 September 2022) allows the visualization of complex networks. The web tool shows protein-protein associations according to different types of known interactions, for instance, created databases, experiments, or predicted interactions, such as gene neighborhood, gene fusion, or co-occurrence. In addition, other interactions can be visualized and produced by text-mining or protein homology. String nodes represent proteins, and edges represent direct and indirect interactions between proteins. Interesting edges are indicated by dashed lines. Each node’s color represents a different protein of the network; edge colors represent different kinds of direct and indirect interactions: known interactions (light blue and pink), predicted interactions (green, red and blue), and others (text-mining, co-expression, protein homology).

### 4.6. miRNA-Target Prediction of Mif-Csn-Nf-kB Network

Predictions of miRNA–RNA target interactions were performed through the miRNA-target interaction predictor [22,49]. The algorithm has a combined approach that exploits the advantages of artificial neural networks and the influence given by the free energy computation of RNA–RNA binding. A final filter on energy was applied at the end of the analysis, considering only RNA–RNA interactions with energy values < 12 kcal/mol, thus reinforcing the power of the predictions.

### 4.7. miRNA Analysis of Their Evolution Pattern

miRNAs identified by NGS were analyzed to identify their evolution pattern. Different miRNAs were already classified as conserved miRNAs, as they were present in different vertebrate species through evolution as previously showed in [22] (see Table 4 for the complete list of conserved miRNAs). The remaining miRNAs were then analyzed through miRBAse (https://mirbase.org accessed on 6 October 2022) by using Blast alignment. All miRNA sequences were downloaded in FASTA format by using the Ensembl database (https://asia.ensembl.org, accessed on 6 October 2022). Then, each fast sequence was downloaded into the miRBase db to be aligned against different species, to find homologous miRNAs. A miRNA was classified as “conserved” if the following conditions were satisfied: first, at least three species have a homologous miRNA (between human, mouse, worm, fly, arabidopsis); second, the *E*-value < 0.01, third the score of percentage of aligned nucleotide >80%. If none of these conditions is satisfied the analyzed miRNAs were classified as species-specific (Table 5). All detailed analyses are found in Supplementary Material, File S2. 

## Figures and Tables

**Table 1:** Transcripts of *C. robusta* pharynx of differentially expressed genes detected by next-generation sequencing (NGS).

Ensemble ID	Gene Name	Log Fold Change	Down (-)/up (+) Regulation	*p*-Value
ENSCING00000021369	Macrophage migration inhibitory factor-1 (Mif-1)	1.337426682	+	0.005771886
ENSCING00000006933	Nuclear factor kappa-light-chain-enhancer of activated B cells (Nf-kB)	−1.171509012	-	0.011439532
ENSCING00000009525	Eukaryotic translation initiation factor 5-like (If5-like)	−1.041407587	-	0.024225277
ENSCING00000020171	Complement component C6	−1.31525517	-	0.004785769
ENSCING00000006967	Apoptosis-inducing factor 2	−4.983320748	-	5.75 × 10^−20^
ENSCING00000005269	Ci-META4 (meta-4)	−4.382519803	-	1.97 × 10^−16^
ENSCING00000014006	Collectin-46-like	−3.936841979	-	3.62 × 10^−9^
ENSCING00000005686	Complement component C6-like	−3.712293984	-	3.00 × 10^−13^
ENSCING00000013919	Cytochrome P450 2C13, male-specific-like	−3.458407214	-	6.28 × 10^−12^
ENSCING00000001811	E-selectin-like	−1.841338282	-	9.61 × 10^−5^
ENSCING00000002203	Heat shock 70 kDa protein 4L	−1.682141547	-	0.000361039
ENSCING00000008644	T-box transcription factor Ci-mT (mT)	2.266940329	+	0.000455334
ENSCING00000023704	TNF receptor-associated factor 3-like	2.326276709	+	7.85 × 10^−5^
ENSCING00000007413	L-rhamnose-binding lectin CSL3-like	1.770298473	+	0.000345541
ENSCING00000019232	Interleukin 17-1 (il-17-1)	1.611522116	+	0.000760084
ENSCING00000005903	Homeobox transcription factor Hox1 (hox1)	1.511849854	+	0.002721531
ENSCING00000003820	Immunoglobulin superfamily containing leucine-rich repeat protein 2-like	1.57489592	+	0.001058269
ENSCING00000016336	Integrin alpha-V like	1.591865774	+	0.000862848

**Table 2:** Transcripts of *C. robusta* pharynx immune genes detected by next-generation sequencing (NGS) analyses and linked to the Mif-Csn-Nf-kB signaling network.

ENSCINGENE	Gene Name	Protein ID
ENSCING00000015215	4-hydroxyphenylpyruvate dioxygenase(LOC100185199)	Hpd
ENSCING00000005686	heat shock protein 70(hsp70)	Hsp70
ENSCING00000013244	Toll-like receptor 2(ci-tlr2)	Tlr2
ENSCING00000017616	myeloid differentiation primary response protein MyD88(LOC100179208)	Myd88
ENSCING00000004105	mitogen-activated protein kinase kinase(mapkk1/2)	Mapk1/2
ENSCING00000007067	mitogen-activated protein kinase(erk1/2)	Erk1/2
ENSCING00000005640	IkappaB kinase(ikk-epsilon)	Ikk-epsilon
ENSCING00000016280	interleukin-1 receptor-associated kinase 1-binding protein 1-like(LOC100177072)	Irak4
ENSCING00000003721	B-raf proto-oncogene serine/threonine-protein kinase	Raf-1
ENSCING00000002308	elongation factor 1-gamma-A-like(LOC100187007)	Ef1-ɣ-A
ENSCING00000012434	elongation factor 1-beta-like(LOC100176941)	Ef1-β-A
ENSCING00000012434	elongation factor 1-beta/delta-like	Ef1-β/δ like
ENSCING00000009525	translation initiation factor(if5-1)	If-5-like
ENSCING00000021423	macrophage migration inhibitory factor-like(LOC100175627)	Mif-2
ENSCING00000021369	macrophage migration inhibitory factor-like(LOC100183461)	Mif-1
ENSCING00000018967	NF-kappa-B inhibitor zeta-like(LOC100182428)	Nf-kB inhib. zeta like
ENSCING00000006933	Nf-kB protein(nfkbp105)	Nf-kB
ENSCING00000008364	aspartate aminotransferase, cytoplasmic-like(LOC100184965)	Got-1
ENSCING00000003669	aspartate aminotransferase, mitochondrial(LOC100179315)	Got-2
ENSCING00000006501	cullin-1-like(LOC100177902)	Cul-1
ENSCING00000002219	cullin-2(LOC100183130)	Cul-2
ENSCING00000002955	cullin-3(LOC100177000)	Cul-3
ENSCING00000001234	cullin-4A(LOC100175879)	Cul-4A
ENSCING00000019895	F-box/LRR-repeat protein 8-like(LOC100187209)	Fbxl
ENSCING00000007306	cullin-associated NEDD8-dissociated protein 1(LOC100177977)	Cand-1
ENSCING00000022721	RING-box protein 1(LOC100182516)	Rbx-1
ENSCING00000001372	RING-box protein 2-like(LOC100181702)	Rbx-2
ENSCING00000009475	COP9 signalosome complex subunit 1(LOC100176942)	Csn1
ENSCING00000008702	COP9 signalosome complex subunit 2-like(LOC100177051)	Csn2
ENSCING00000002866	COP9 signalosome complex subunit 4-like(LOC100183592)	Csn4
ENSCING00000006436	COP9 signalosome complex subunit 5-like(LOC100187322)	Csn5
ENSCING00000000624	COP9 signalosome complex subunit 6(LOC100176116)	Csn6
ENSCING00000021617	COP9 signalosome complex subunit 7a-like(LOC100184522)	Csn7A-like
ENSCING00000011567	COP9 signalosome complex subunit 8-like(LOC100182035)	Csn8-like

**Table 3:** BLAST analysis of the Csn complex, cullin family, Rbx proteins, Cand-1, and Fbxl between *C. robusta* and *H. sapiens*. The table shows the percentage of identity between *C. robusta* and *H. sapiens* protein sequences and the protein domains identified.

Protein Name	*C. robusta* Protein ID	*H. sapiens* Protein ID	Sequence Identity	Protein Domain
Cul-1	F6PW28	Q13616	72.9%	CULLIN-DOMAIN
Cul-2	F6Z726	Q13617	46.7%	CULLIN-DOMAIN
Cul-3	F6R472	Q13618	71.2%	CULLIN-DOMAIN
Cul-4A	F6SBC7	Q13619	62.7%	CULLIN-DOMAIN
Csn-1	F7AEY7	Q13098	59.3%	PCI-DOMAIN
Csn-2	F6WYI9	P61201	80.9%	PCI-DOMAIN
Csn-4	F6TMN3	Q9BT78	68.5%	PCI-DOMAIN
Csn-5	F6QLM7	Q92905	80.2%	MPN-DOMAIN
Csn-6	F6RRF2	Q7L5N1	58.2%	MPN-DOMAIN
Csn7a-like	F6Z0V4	Q9UBW8	45.8%	PCI-DOMAIN
Csn8-like	F6TBA1	Q99627	35.4%	PCI-DOMAIN
Rbx-1	H2XNZ7	P62877	92.2%	RING-TYPE DOMAIN
Rbx-2	F6SAA4	Q9UBF6	82.6%	RING-TYPE DOMAIN
Cand-1	F6Z3T2	Q86VP6	56.3%	HEAT REPEAT
Fbxl	H2XKE5	Q96CD0	24.6%	F-BOX DOMAIN

**Table 4:** Conserved miRNAs linked to inflammation processes that have been evidenced in the *C. robusta* transcriptome by NGS and that have been identified by the MiRNATIP algorithm. Where “none” is present, no literature information related to inflammation is available.

miRNA ID	Conserved miRNAs Evidenced by *C. robusta* NGS	Authors	Reference
let-7a	ENSCING00000017776	Yu et al.	[23]
let-7b	ENSCING00000017757	Teng et al.	[24]
let-7c	ENSCING00000017755	Zhao et al.	[25]
miR-92a	ENSCING00000017761	Li et al.	[26]
miR-92c	ENSCING00000017777	None	None
miR-126	ENSCING00000017730	Poissonnier et al.	[27]
miR-141	ENSCING00000017782	Lin et al.	[28]

**Table 5:** Species-specific miRNAs detected by NGS that have been identified by the MiRNATIP algorithm.

miRNA ID	Species-Specific miRNAs Evidenced by *C. robusta* NGS	miRNA ID	Species-Specific miRNAs Evidenced by *C. robusta* NGS
cin-miR-4011-a	ENSCING00000021193	cin-miR-4163	ENSCING00000020782
cin-miR-4019	ENSCING00000025043	cin-miR-4183	ENSCING00000021879
cin-miR-4024	ENSCING00000021882	cin-miR-4186	ENSCING00000020139
cin-miR-4034	ENSCING00000018691	cin-miR-4187	ENSCING00000024515
cin-miR-4053	ENSCING00000020571	cin-miR-4189	ENSCING00000018460
cin-miR-4056	ENSCING00000021074	cin-miR-4197	ENSCING00000023707
cin-miR-4069	ENSCING00000021942	cin-miR-4200	ENSCING00000021921
cin-miR-4075	ENSCING00000025167	cin-miR-5596b	ENSCING00000020725
cin-miR-4089	ENSCING00000020134	cin-miR-5598	ENSCING00000021178
cin-miR-4094	ENSCING00000022222	cin-miR-5600	ENSCING00000017949
cin-miR-4098	ENSCING00000021981	cin-miR-5605	ENSCING00000023578
cin-miR-4109	ENSCING00000022889	cin-miR-5609	ENSCING00000024461
cin-miR-4144	ENSCING00000019473	cin-miR-5611	ENSCING00000023047

## Supplementary Materials

The following supporting information can be downloaded at: https://www.mdpi.com/article/10.3390/ijms24044112/s1.
